# Synthesis, Crystal Structure and Anti-Fatigue Effects of Some Benzamide Derivatives

**DOI:** 10.3390/molecules19011034

**Published:** 2014-01-16

**Authors:** Xianglong Wu, Wutu Fan, Yalei Pan, Yuankun Zhai, Yinbo Niu, Chenrui Li, Qibing Mei

**Affiliations:** 1 Key Laboratory for Space Bioscience and Biotechnology, School of Life Sciences, Northwestern Polytechnical University, Xi’an 710072, China; 2 Department of Pharmacology, School of Pharmacy, Fourth Military Medical University, Xi’an 710032, China

**Keywords:** benzamide derivatives, 1-BCP, X-ray diffraction, synthesis, anti-fatigue effects

## Abstract

A series of benzamide derivatives such as 1-(1,3-benzodioxol-5-ylcarbonyl) piperidine (1-BCP) were synthesized by the reaction of substituted benzoic acids with piperidine, morpholine or pyrrolidine using a novel method. The crystals of these benzamide derivatives were obtained by recrystallization. Structures of target and intermediate compounds were determined *via* FT-IR, ^1^H-NMR and elemental analysis and X-ray crystallography of select examples. The crystal structures of these compounds have potential applications to identify the binding site for allosteric modulators of the α-amino-3-hydroxy-5-methylisoxazole-4-propionic acid (AMPA) receptor. The anti-fatigue effects of the benzamide derivatives in weight-loaded forced swimming mice were investigated in a swimming endurance capacity test used as an indicator of fatigue. The swimming times to exhaustion were longer in the **b3**, **d3**, and **e3** groups than in the caffeine group (*p <* 0*.*05). In conclusion, **b3**, **d3** and **e3** enhanced the forced swimming capacity of mice. The mechanism of the anti-fatigue effects will be studied in the future.

## 1. Introduction

Ionotropic glutamate receptors are the major excitatory amino acid neurotransmitter receptors in the vertebrate central nervous system (CNS). There are three functionally distinct receptor subclasses: α-amino-3-hydroxy-5-methyl-4-isoxazolepropionic acid (AMPA), kainate and N-methyl-D-aspartate (NMDA) [1]. AMPA receptors mediate most of the fast excitatory amino acid transmission in the CNS [2,3]. Attention has focused on drugs that modulate AMPA receptors because of their potential to enhance memory and treat certain pathologies [4]. Positive allosteric modulators of AMPA may have an advantage over direct-acting agonists or compounds that promote glutamate release, in that such drugs could increase glutamatergic tone, without the obvious liability for direct receptor-mediated excitotoxicity [5].

**Figure 1:**
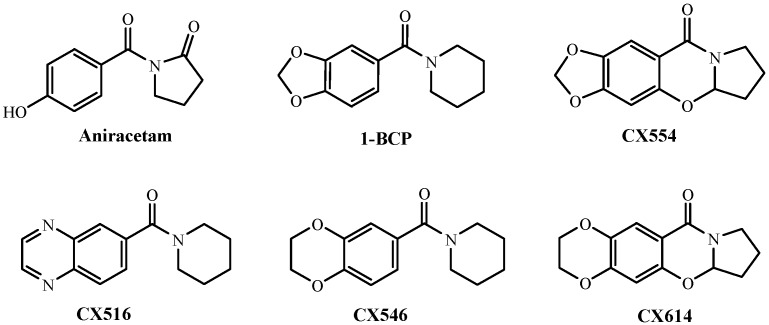
Chemical structures of ampakines.

Ampakines are small benzamide compounds that are positive allosteric modulators of AMPA receptors in the mammalian brain. Ampakines were initially derived from the nootropic drug aniracetam which was discovered by Ito *et al.* [6]. Several ampakine compounds (see Figure 1), including 1-BCP, CX516, CX554, CX546 and CX614 can facilitate synaptic plasticity and improve learning and memory in both animals and humans [7].

These compounds contain as common element a benzamide core, usually in the form of a benzoylpiperidine or a benzoylpyrrolidine. They play a potential therapeutic role in the treatment of neurodegenerative disorders, cognitive impairment, depression, Alzheimer’s disease and schizophrenia [8]. They have also been shown to be effective in animal models to treat various neuronal disorders [9] and to enhance viability of neurons [10], probably by promoting expression of growth factors [11].

In this paper, we synthesized several benzamide derivatives which contain a benzamide core and studied their unexpected anti-fatigue activity in the laboratory. To this end we first investigated their anti-fatigue activity by weight-loaded forced swimming tests using a mouse model.

Numerous methods have been developed toward the synthesis of benzamide derivatives. Formation of the amide bond has been one of the most widely studied reactions in organic chemistry [12]. It has been taken for granted that amide bond formation is easily realized by reacting an activated carboxylic acid with an amine.

Generally, most amides are prepared by the condensation of carboxylic acids and amines in the presence of dehydrating reagents, such as B(OMe)_3_, TsCl/Py, TiCl_4_, Ph_2_POCl, Ph_3_P/PySSPy, *N,N'*-dicyclohexylcarbodiimide (DCC), cyanuric chloride, 2-halo-*N*-methylpyridinium, and (benzotriazol-1-yl) oxy-tris(dimethylamino)phosphonium hexafluorophosphate (BOP), or the carbonylation of aryl halides with amines in the presence of Pd(OAc)_2_, and CO, *etc.* [13]. These methods all have some advantages and disadvantages.

As a result, we report in this paper a convenient approach for the synthesis of amides. Substituted benzoic acids were first treated with N-hydroxysuccinimidyl trifluoroacetate (NHS-TFA). Then, the resulting intermediates were treated with piperidine, morpholine or pyrrolidine to give the target compounds. Unexpectedly, crystals of the intermediate or target compounds were also obtained. All compounds were analyzed via FT-IR, ^1^H-NMR and elemental analysis. The anti-fatigue activity of the benzamide derivatives in weight-loaded forced swimming mice was then investigated. Detailed results and discussion are elaborated in the following sections.

## 2. Results and Discussion

### 2.1. Synthesis and Structural Confirmation

Benzamide derivatives are typically prepared by chlorination of benzoic acids followed by amidation of the resulting acid chlorides with an amine compound. Although the purified product can be obtained by flash column chrmatography, difficulties can’t be avoided if using this method [14]. 1-BCP was also prepared by the reaction of piperonaldehyde with an equimolar amount of piperidine at 100 °C in the presence of RuH_2_(PPh_3_)_4_ catalyst [15]. A specific catalyst was used in this method. Beller and co-workers used CataCxium A at 80–130 °C and 2 bar CO pressure as the ligand for primary amide synthesis from various substituted aryl bromides and heteroaryl bromides [16]. Recently, palladium-based homogeneous catalysts were also reported for substrates such as benzyl chlorides and aryl chlorides [17].

**Scheme 1:**
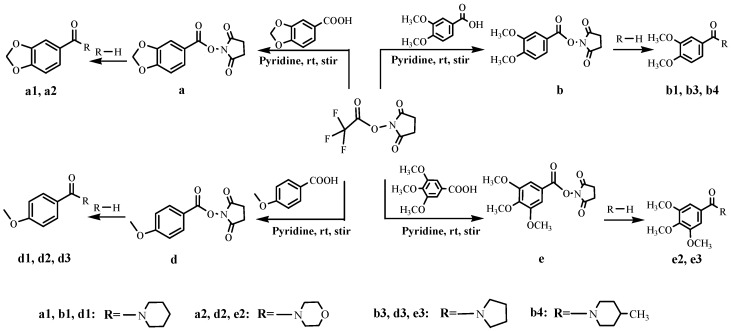
Synthesis of benzamide derivatives.

In this paper, piperonylic acid was treated with *N*-hydroxysuccinimidyl trifluoroacetate (NHS-TFA) at room temperature (Scheme 1). Then, the intermediate was reacted with piperidine to give the target compound 1-BCP, which could be easily purified by recrystallization from ethanol. The approach provided an efficient and environmentally friendly pathway to synthetic benzamide compounds [18] in good to excellent yields. A total of ten benzamide derivatives were synthesized. The compounds were characterized by elemental analysis, FT-IR, ^1^H-NMR and X-ray crystallography. The information provided should be useful in the area of medical or pharmaceutical applications. 

### 2.2. Crystal Structures

**Figure 2:**
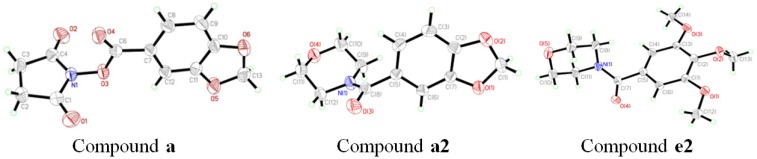
ORTEP drawings of molecules **a**, **a2** and **e2** with 30% probability displacement ellipsoids.

**Figure 3:**
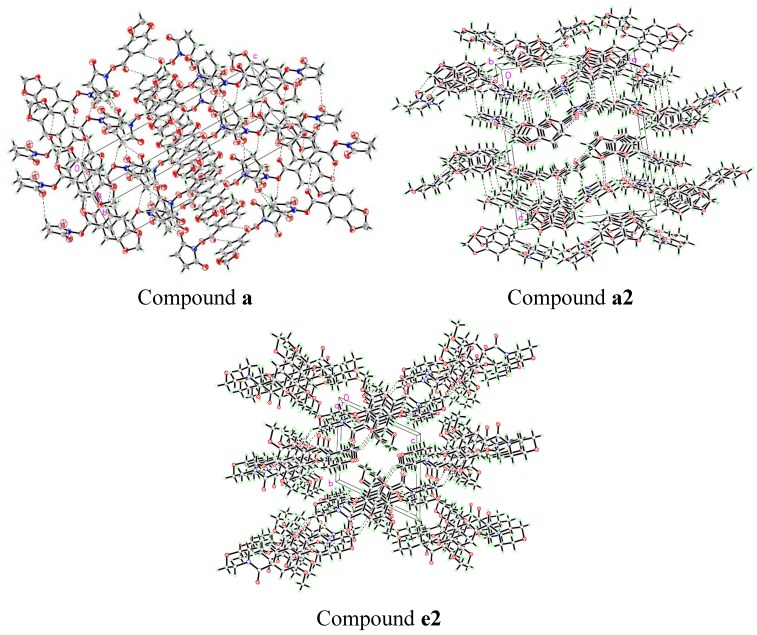
View of the molecular packing in **a**, **a2** and **e2**.

Single crystals of compounds **a**, **a2** and **e2** were obtained by recrystallization and their structures were further confirmed by X-ray diffraction determination. The data of compounds **a**, **a2** and **e2** are deposited as CCDC Nos. 815292, 960811 and 960813, respectively. The perspectives and packing views are shown in Figure 2 and Figure 3, respectively. The crystal data, details concerning data collection and structure refinement are listed in Table 1.

**Table 1:** Crystal data and structure refinement for **a**, **a2** and **e2**.

	a	a2	e2
CCDC Deposit No.	815292	960811	960813
Empirical formula	C_12_H_9_NO_6_	C_12_H_13_NO_4_	C_14_H_19_NO_5_
Formula weight	263.20	235.23	281.30
Temperature (K)	296(2)	296(2)	296
Wavelength (Å)	0.71073	0.71073	0.71073
Crystal system	Monoclinic	Orthorhombic	Triclinic
Space group	P2(1)/n	P bca	P-1
a (Å)	8.7694(11)	14.595(3)	8.6577(9)
b (Å)	6.2631(8)	7.7849(14)	9.5460(10)
c (Å)	22.168(3)	20.112(4)	9.8578(10)
α (°)	90	90	64.1760(10)
β (°)	94.814(2)	90	77.037(2)
γ (°)	90	90	71.6540(10)
Volume (Å^3^)	1213.3(3)	2285.1(7)	692.44(12)
Z	4	8	2
D_calc_ (g/cm^3^)	1.441	1.368	1.349
Abs.coefficient (mm^−1^)	0.118	0.104	0.096
F(000)	544	992	300
Crystal size (mm^3^)	0.14 × 0.11 × 0.10	0.20 × 0.10 × 0.10	0.39 × 0.27 × 0.20
θ limit (°)	1.84 to 25.10	2.46 to 25.00	2.31 to 25.00
Ranges/indices *h. k. l.*	−10/10, −7/6, −26/26	−15/17, −9/9, −23/17	−9/10, −9/11, −11/11
Refinement method	Full-matrix least-squares on F^2^		
Reflections collected/ unique/R_int_	5777 / 2165/ 0.043	10446/2001/0.033	3490/ 2432/0.0205
Completeness to θ = 25.10°	99.6%	99.6%	99.3%
Data/restraints/parameters	2165/0/173	2001/0/155	2432/0/185
Goodness of fit on F^2^	1.088	1.023	1.085
R_1_, wR_2_	0.0456, 0.1199	0.0589, 0.1553	0.0404, 0.1261
Extinction coefficient	0.020(4)	0.0028(11)	0.120(12)
Largest diff. peak and hole(e. Å ^−3^)	0.149 and −0.161	0.489 and −0.219	0.155 and −0.186

The crystals of 3-benzodioxole-5-carboxylic acid 2,5-dioxo-1-pyrrolidinyl ester (**a**) are monoclinic, space group P2(1)/n with four molecules per unit cell. The O (6) atom lies within the mean plane of the benzene, as indicated by O(6)-C(10)-C(11)-C(12) torsion angle of −179.16°. The O(5) atom is also in the mean plane of the benzene, as indicated by the O(6)-C(10)-C(11)-O(5) torsion angle of 0.6(2)°. All carbon and oxygen atoms of piperonyl are in a plane. The succinimide ring also forms a plane. The dihedral angle between the mean plane of the piperonyl and succinimide rings is 74.69°. In the crystal packing, as shown in Figure 3, weak intermolecular hydrogen bonds are observed (showed as dashed lines).

The crystals of compound **a2** are orthorhombic, space group Pbca with eight molecules per unit cell. The torsion angles of C(12)-N(1)-C(9)-C(10) and C(11)-O(4)-C(10)-C(9) [53.7(3)° and 58.5(3)°, respectively] show that the morpholine ring is in a typical chair conformation. The dihedral angle between the mean plane of benzene and a plane formed by N(1), C(9) and C(12) is 89.8°. In the crystal packing, as shown in Figure 3, adjacent molecules are crossed stacked through strong offset π···π aromatic stacking interactions. Intermolecular hydrogen bonds are also observed.

The crystals of compound **e2** are triclinic, space group P-1 with two molecules per unit cell. The morpholine ring is also is a typical chair conformation. In the crystal packing, as shown Figure 3 intermolecular hydrogen bonds are also observed (showed as dashed lines).

### 2.3. Effect on Forced Swimming Capacity

**Figure 4:**
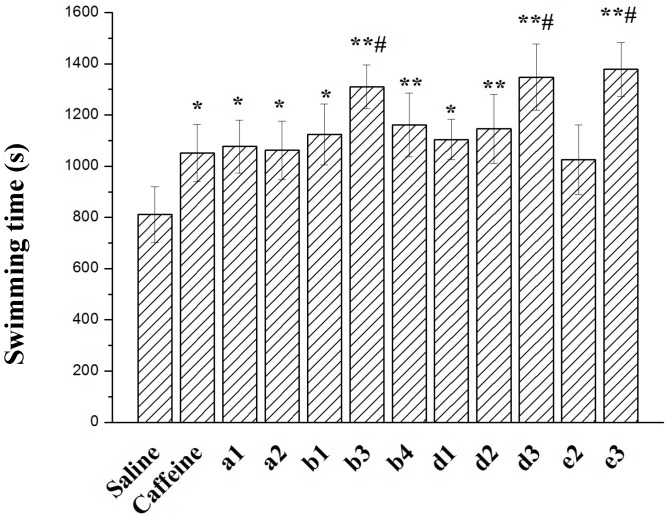
Effects of compounds on the forced swimming time. ** *p* < 0.01 compared with the saline group, * *p* < 0.05 compared with the saline group, # *p* < 0.05 compared with the caffeine group.

The forced swimming capacities are shown in Figure 4. There are significant differences in the swimming time to exhaustion between the control group and each treatment group. The swimming times to exhaustion of the caffeine (positive control group), **b3**, **d3**, and **e3** groups were 1052 ± 148 s, 1310 ± 85 s, 1348 ± 139 s, and 1378 ± 105 s, respectively. Thus, the swimming times to exhaustion of the **b3**, **d3**, and **e3** groups were significantly longer than those of the caffeine group (*p* < 0.05). It is worthwhile to note that **b3**, **d3**, and **e3** have a common pyrrolidine ring. But what is the real reason? Further observation and study are needed. On the other hand, the swimming times to exhaustion of the **a2**, **b1**, **b4**, **d1**, **d2**, and **e2** groups were longer than that of the normal saline group. However, there was no significant difference in between the caffeine group and each of the other treatment groups.

## 3. Experimental

### 3.1. General Information

Melting points were taken on a X-4 micro melting point apparatus and are uncorrected. ^1^H-NMR spectra were recorded out in CDCl_3_ on a Varian Inova-400 spectrometer and chemical shifts were reported relative to internal Me_4_Si. Infrared spectra were obtained as KBr pellets on an Equiox-55 FTIR spectrometer. Elemental analyses were performed with a Vario EL-III instrument. The X-ray diffraction data were collected on a Bruker SMART AREX II CCD diffractometer equipped with a graphite monochromated Mo *Kα* radiation (λ = 0.71073 Å) source by using the ω-2θ scan technique at room temperature. The structure was solved by direct methods with SHELXS-97 [19], and refined using SHELXL-97 [20]. Hydrogen atoms were generated geometrically. Molecular illustrations were prepared using the XP package [21]. CCDC 815292 (for compound **a**), 960811 (for compound **a2**) and 960813 (for compound **e2**) contain the supplementary crystallographic data for this paper. These data can be obtained free of charge from http://www.ccdc.cam.ac.uk/conts/retrieving.html (or from the Cambridge Crystallographic Data Centre, 12, Union Road, Cambridge CB2 1EZ, UK; fax: +44 1223 336033).

### 3.2. Synthesis

#### 3.2.1. Preparation of *N*-Hydroxysuccinimidyl Trifluoroacetate (NHS-TFA)

**Scheme 2:**
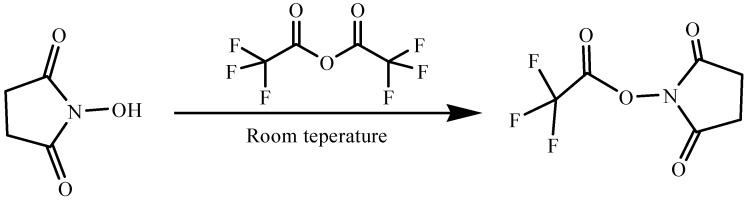
Preparation of N-hydroxysuccinimidyl trifluoroacetate.

The synthesis of NHS-TFA is shown in Scheme 2. To trifluoroacetic anhydride (15 ml, 71.2 mmol), *N*-hydroxysuccinimide (5 g, 42.6 mmol) was slowly added with vigorously stirring. On a larger scale, a condenser equipped with a drying tube may be needed. The solution was stirred at room temperature about 4 h until the reaction was completed. The reaction solution was evaporated *in vacuo* to remove trifluoroacetic anhydride and trifluoroacetic acid to give a thick oil. Then, it was further dried below 70 °C until a free-flowing colorless solid was obtained. The product is of good quality if it gives bubbles when dissolved in methanol.

#### 3.2.2. Synthesis of 3-Benzodioxole-5-carboxylic acid, 2,5-dioxo-1-pyrrolidinyl Ester (**a**)

Piperic acid (1.66 g, 10 mmol) was dissolved in anhydrous THF (50 mL) followed by the addition of anhydrous pyridine (4 mL) and N-hydroxysuccinimidyl trifluoroacetate (NHS-TFA) (6.33 g, 30 mmol). The reaction mixture was stirred at room temperature for 4 h. The reaction mixture was concentrated under reduced pressure. The residue was dissolved in CH_2_Cl_2_ (80 mL) and washed with 1mol/L HCl (3 × 80 mL), followed by saturated brine (3 × 80 mL). The organic layer was dried over anhydrous Na_2_SO_4_, filtered and concentrated to give 3-benzodioxole-5-carboxylic acid, 2,5-dioxo-1-pyrrolidinyl ester (**a**). Single crystals of (**a**) for X-ray diffraction experiments were grown from ethanol-water mixture (1:1). White solid; Yield: 88%; m.p. 152–154 °C; FTIR (cm^−1^): 3103, 2995, 2913, 1771, 1730, 1623, 1485, 1442, 1375, 1263, 1210, 1073, 994, 830; ^1^H-NMR (CDCl_3_) δ: 2.91 (s, 4H), 6.09 (s, 2H), 6.90 (d, 1H), 7.51 (s, 1H), 7.76 (d,1H); Anal. calcd. For C_12_H_9_NO_6_: C, 54.76; H, 3.45; N, 5.32; Found C, 54.72; H, 3.41; N, 5.27.

#### 3.2.3. Synthesis of 3,4-Dimethoxybenzoic acid, 2,5-dioxo-1-pyrrolidinyl ester (**b**)

Compound **b** was prepared from 3,4-dimethoxybenzoic acid (1.82 g, 10 mmol) and NHS-TFA (6.33 g, 30 mmol) by the procedure utilized for compound **a**. Compound **b** was obtained as a white solid in 84% yield. FTIR (cm^−1^): 3094, 3023, 2940, 2841, 1734, 1599, 1513, 1458, 1448, 1417, 1373, 1273, 1206, 1143, 1071, 1017, 878, 810; ^1^H-NMR (CDCl_3_) δ: 2.92 (s, 4H), 3.94 (s, 6H), 6.94 (d, 1H, *J* = 8.8 Hz), 7.57 (s, 1H), 7.82 (d, 1H, *J* = 6.8 Hz); Anal. calcd. For C_13_H_13_NO_6_: C, 55.91; H, 4.69; N, 5.02; Found C, 55.89; H, 4.67; N, 5.04.

#### 3.2.4. Synthesis of 4-Methoxybenzoic acid, 2,5-dioxo-1-pyrrolidinyl ester (**d**)

This compound was prepared from 4-methoxybenzoic acid (1.52 g, 10 mmol) and NHS-TFA (6.33 g, 30 mmol) by the procedure utilized for compound **a**. Compound (**d**) was obtained as a white solid in 86% yield. m.p. 142–144 °C; FTIR (cm^−1^): 3433, 3111, 2940, 2848, 1766, 1735, 1602, 1514, 1457, 1427, 1377, 1271, 1255, 1216, 1183, 1073, 1022, 984, 847, 757; ^1^H-NMR (CDCl_3_) δ: 2.89 (s, 4H), 3.89 (s, 3H), 6.97 (d, 2H, *J* = 8.8 Hz), 8.09 (d, 2H, *J* = 8.8 Hz). 

#### 3.2.5. Synthesis of 3,4,5-Trimethoxybenzoic acid, 2,5-dioxo-1-pyrrolidinyl Ester (**e**)

This compound was prepared from 3,4,5-trimethoxybenzoic acid (2.12 g, 10 mmol) and NHS-TFA (6.33 g, 30 mmol) by the procedure utilized for compound **a**. Compound (**e**) was obtained as white solid in 80% yield. m.p. 125–127 °C; FTIR (cm^−1^): 2997, 2952, 1763, 1734, 1588, 1504, 1469, 1422, 1339, 1260, 1241, 1203, 1166, 1128, 1078, 989, 892, 750; ^1^H-NMR (CDCl_3_) δ: 2.91(s, 4H), 3.92(s, 9H), 7.38(s, 2H).

#### 3.2.6. Synthesis of Compounds **a1** and **a2**

3-Benzodioxole-5-carboxylic acid, 2,5-dioxo-1-pyrrolidinyl ester (2.63 g, 10 mmol) was dissolved in CH_2_Cl_2_ (50 mL) followed by the addition of piperidine (2 mL). The resultant mixture was stirred at room temperature for 2 h. The mixture was washed with 1 mol/L HCl (3 × 80 mL) and saturated brine (3 × 80 mL), and then dried over anhydrous Na_2_SO_4_. The solvent was removed under reduced pressure and the residue was crystallized from ethanol as needles. The compound **a1** was obtained as a white powder. Compound **a2** were prepared according to the same procedure. Single crystals of compound (**a2**) for X-ray diffraction experiments were grown from ethanol.

*N-(3,4-methylenedioxybenzoyl) piperidine* (**a1**). Needle-like solid; Yield: 83%; m.p. 51–52 °C (lit. [22]: 51–52 °C); FTIR (cm^−1^): 3071, 3006, 2926, 2853, 1713, 1607, 1445, 1282, 1248, 1077, 1034, 929, 802; ^1^H-NMR (CDCl_3_) δ: 1.56–1.67 (m, 6H), 3.43 (t, 4H), 6.00 (s, 2H), 6.80 (d, 1H), 6.89 (s, 1H), 6.92 (d, 1H). Anal. calcd. For C_13_H_15_NO_3_: C, 66.97; H, 6.52; N, 5.97; Found C, 66.94; H, 6.48; N, 6.00.

*N-(3,4-methylenedioxybenzoyl) morpholine* (**a2**). Needle-like solid; Yield: 80%; m.p. 82–84 °C (lit. [23]: 83–84 °C); FTIR (cm^−1^): 3433, 3040, 2974, 2899, 2859, 1620, 1494, 1444, 1368, 1342, 1283, 1246, 1108, 1037,940, 833; ^1^H-NMR (CDCl_3_) δ: 3.69 (s, 8H), 6.00 (s, 2H), 6.83 (d, 1H, *J* = 8.0 Hz), 6.91 (s, 1H), 6.93 (d, 1H, *J* = 8Hz); Anal. calcd. For C_12_H_13_NO_4_: C, 61.27; H, 5.57; N, 5.95; Found C, 61.59; H, 5.52; N, 5.98.

#### 3.2.7. Synthesis of Compounds **b1**, **b3** and **b4**

These compounds were prepared from 3,4-dimethoxybenzoic acid, 2,5-dioxo-1-pyrrolidinyl ester and piperidine (pyrrolidine or 4-methylpyridine) by the procedure utilized for compound **a1**.

*N-(3,4-Dimethoxybenzoyl) piperidine* (**b1**). White solid; Yield: 84%; m.p. 35–36 °C (lit. [24]: 35–37 °C); FTIR (cm^−1^): 3443, 2943, 2855, 1617, 1580, 1518, 1432, 1331, 1267, 1137, 1018, 870; ^1^H-NMR (CDCl_3_) δ: 1.55–1.68 (m, 6H), 3.45–3.65 (m, 4H), 3.86 (s, 6H), 6.86 (d, 1H, *J* = 8.0 Hz), 6.97 (d, 1H, *J* = 8.0 Hz), 6.98 (s, 1H). 

*N-(3,4-Dimethoxybenzoyl) pyrrolidine* (**b3**). Colorless oil; Yield: 83%; FTIR (cm^−1^): 3437, 2972, 2935, 2875, 1612, 1575, 1512, 1454, 1417, 1384, 1328, 1249, 1176, 1120, 1022, 869, 821, 758; ^1^H-NMR (CDCl_3_) δ: 1.87 (t, 2H, *J* = 6.4 Hz), 1.96 (t, 2H, *J* = 6.4 Hz), 3.50 (t, 2H, *J* = 6.0 Hz), 3.64 (t, 2H, *J* = 6.4 Hz), 3.91 (s, 6H), 6.85 (d, 1H, *J* = 8.0 Hz), 7.12 (d, 1H, *J* = 8.8 Hz), 7.14 (s,1H). 

*(3,4-Dimethoxyphenyl)(4-methyl-1-piperidinyl) methanone* (**b4**). Colorless oil; Yield: 82%; FTIR (cm^−1^): 3444, 2957, 2863, 1631, 1517, 1437, 1324, 1271, 1141, 1072, 818; ^1^H-NMR (CDCl_3_) δ: 0.98 (d, 3H, *J* = 5.6 Hz), 1.15–1.24 (m, 2H), 1.61–1.79 (m, 4H), 3.90 (s, 6H,), 6.85 (d, 1H, *J* = 8.0 Hz), 6.97 (d, 1H, *J* = 8.4 Hz), 6.98 (s, 1H).

#### 3.2.8. Synthesis of Compound **d1**–**d3**

These compounds were prepared from 4-methoxybenzoic acid, 2,5-dioxo-1-pyrrolidinyl ester and piperidine (morpholine or pyrrolidine) by the procedure utilized for compound **a1**. 

*N-(4-Methoxybenzoyl) piperidine* (**d1**). Colorless oil; Yield: 80%; ^1^H-NMR (CDCl_3_) δ: 1.58–1.67 (m, 6H), 3.30–3.71 (m, 4H), 3.83 (s, 3H), 6.90 (d, 2H, *J* = 8.4 Hz), 7.37 (d, 2H, *J* = 8.4 Hz). The properties match those reported in the literature [25]. 

*N-(4-Methoxybenzoyl) morpholine* (**d2**). Colorless oil. Yield: 82%; FTIR (cm^−1^): 3427, 3188, 2931, 2859, 1764, 1690, 1645, 1610, 1494, 1459, 1321, 1254, 1229, 1168, 1109, 1051, 1028, 838, 757, 706; ^1^H-NMR (CDCl_3_) δ: 3.69 (s, 4H), 3.84 (s, 3H), 6.92 (d, 2H, *J* = 8.4 Hz), 7.39 (d, 2H, *J* = 8.4 Hz). The properties match those reported in the literature [13]. 

*N-(4-Methoxybenzoyl) pyrrolidine* (**d3**). White solid; Yield: 85%; m.p. 76–77 °C (lit. [26]: 78.4–78.8 °C); FTIR (cm^−1^): 3521, 3111, 3082, 2847, 1766, 1735, 1602, 1514, 1457, 1427, 1377, 1271, 1255, 1217, 1183, 1074, 1022, 984, 847, 757; ^1^H-NMR (CDCl_3_) δ: 1.85-1.90 (m, 2H), 1.92–1.97 (m, 2H), 3.48 (t, 2H, *J* =6.4 Hz), 3.65 (t, 2H, *J* = 6.8 Hz), 3.84 (s, 3H), 6.90 (d, 2H, *J* = 8.8 Hz), 7.52 (d, 2H, *J* = 8.8 Hz).

#### 3.2.9. Synthesis of Compounds **e2** and **e3**

Compounds **e2** and **e3** were prepared from 3,4,5-trimethoxybenzoic acid, 2,5-dioxo-1-pyrrolidinyl ester and morpholine or pyrrolidine by the procedure utilized for compound **a1**. Single crystals of compound **e2** for X-ray diffraction experiments were grown from ethanol. 

*N-(3,4,5-Trihydroxybenzoyl) morpholine* (**e2**). White solid; Yield: 79%; m.p. 97–99 °C (lit. [12]: 96–98 °C); FTIR (cm^−1^): 3430, 2974, 2934, 2871, 1765, 1637, 1586, 1449, 1416, 1327, 1233, 1132, 958, 854; ^1^H-NMR (CDCl_3_) δ: 3.69–3.71 (m, 4H), 3.87 (s, 9H), 6.63 (s, 2H).

*N-(3,4,5-Trihydroxybenzoyl) pyrrolidine* (**e3**). White solid; Yield: 84%; m.p. 182–183 °C. (lit. [27]: 183–186 °C); ^1^H-NMR (CDCl_3_) δ: 1.89 (dd, 2H, *J* = 7.4 Hz, 6.0 Hz), 1.97 (dd, 2H, *J* = 6.8 Hz, 6.4 Hz), 3.46 (t, 2H, *J* = 6.8 Hz), 3.64 (t, 2H, *J* = 6.8 Hz), 3.86 (s, 9H), 6.75 (s, 2H).

### 3.3. Anti-Fatigue Activity Assessment of Benzamide Derivatives

#### 3.3.1. Animals

Six-week-old male Kunming mice (18–22 g, specific pathogen-free grade, SPF) were obtained from the Academy of Experimental Animal Center of The Fourth Military Medical University (Xi’an, China). Mice were maintained under normal conditions: 21–26 °C temperature, 60%–70% relative humidity, 12:12 h dark/light cycle, and free access to laboratory standard diet and water. All mice were quarantined and adapted for 7 days after arriving. All animal experiments were conducted under institutional guidelines and approved by the Ethical Committee for Animal Care and Use of the Fourth Military Medical University.

#### 3.3.2. Anti-Fatigue Activity

The anti-fatigue activity of benzamide derivatives was evaluated by the weight loaded swimming model in mice. The model is a reliable measure of anti-fatigue treatment as established in both laboratory animals and humans [28,29,30]. All mice were randomly divided into 12 groups, two control groups and ten treatment groups, seven mice per group. Benzamide derivatives were dissolved in carboxymethylcellulose sodium (CMC) 5 g/L aqueous solution, respectively. One control group received the same volume of saline solution. Another control group received caffeine as a positive control group. Benzamide derivatives were given to mice at concentrations of 0.1 mmol/kg body weight. Samples were orally administered into mice using a feeding atraumatic needle, once per day at 1:00–3:00 pm for one week. After each treatment, all groups of the mice were allowed to rest 30 min and were forced to swim for ten minutes to become accustomed to swimming. The size of swimming pool was designed as 50 cm × 50 cm × 40 cm, filled with fresh water at 30 ± 2 °C. A tin wire (5% of body weight) was loaded on the tail root of the mouse. The mice were assessed to be exhausted when they failed to rise to the surface of water to breathe within a 7 s period [31]. At the end of the session, the mice were removed from the water, dried with a paper towel, and placed back in their home cages. Water in the container was drained after each session. The swimming time to exhaustion was used as the index of the forced swimming capacity. 

#### 3.3.3. Statistical Analysis

Data were analyzed using SPSS 13.0 version. The results were expressed as the mean ± S.E.M. The data were subjected to one-way analysis of variance (ANOVA) followed by Tukey–Kramer *post-hoc* analysis. The level of *p* < 0.05 was used as the criterion of statistical significance.

## 4. Conclusions

In the current study, we have established for the first time a rapid and highly efficient method for the synthesis of benzamide derivatives. The structures of the compounds have been determined by FTIR and ^1^H-NMR spectroscopy, elemental analysis and X-ray diffraction of select examples (compounds **a**, **a2** and **e2)**. The crystal structures have potential applications in identifying the binding site for allosteric modulators of the AMPA receptor, and should be useful in the area of medical or pharmaceutical applications. 

Preliminary studies suggest that compound **b3**, **d3** and **e3** increased swimming time to exhaustion in weight-loaded forced swimming mice. They have potential application in developing new drug candidates with anti-fatigue activity. Because a large number of complex mechanisms may be involved in the exercise-induced fatigue, further research including molecular study is required to evaluate the anti-fatigue mechanism.

## Supplementary Materials

Supplementary File 1
