# Supplementary material for: Synthesis, Crystal Structure and Anti-Fatigue Effects of Some Benzamide Derivatives
Source: Molecules. 2014 Jan 16;19(1):1034–46. doi: 10.3390/molecules19011034 (PMC6271547; doi:10.3390/molecules19011034)
Supplement: Supplementary File 1 [file molecules-19-01034-s001.doc]

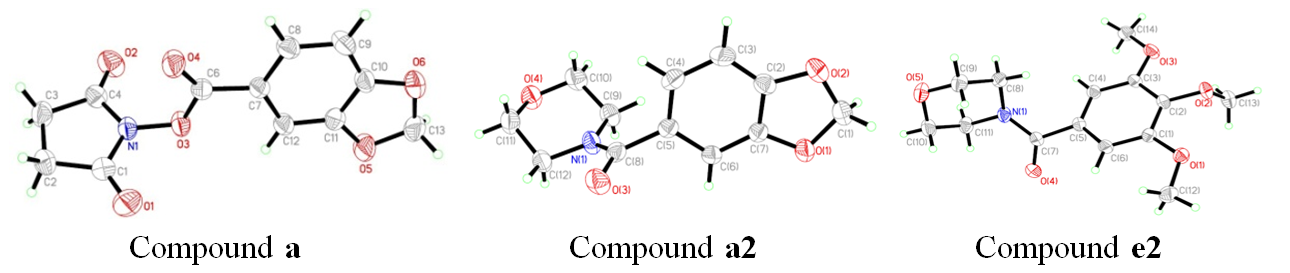


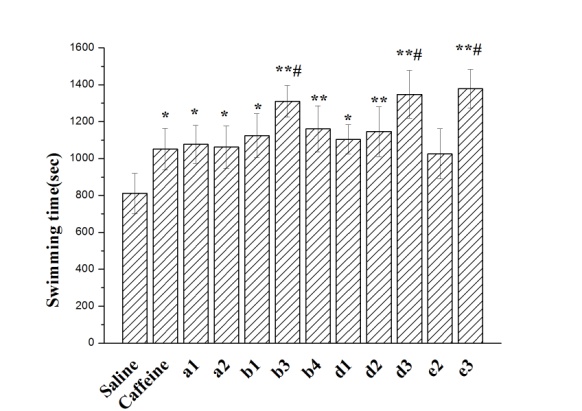
A series of benzamide derivatives were synthesized by the reaction of substituted benzoic acids with piperidine, morpholine or pyrrolidine using a novel method. Structures of target and intermediate compounds were determined *via* FT-IR, ^1^H-NMR and elemental analysis and X-ray crystallography of select examples. The anti-fatigue effects of the benzamide derivatives in weight-loaded forced swimming mice were investigated in a swimming endurance capacity test used as an indicator of fatigue.
